# A Flexible Pressure Sensor Based on Silicon Nanomembrane

**DOI:** 10.3390/bios13010131

**Published:** 2023-01-12

**Authors:** Lixia Cheng, Xiaojian Hao, Guochang Liu, Wendong Zhang, Jiangong Cui, Guojun Zhang, Yuhua Yang, Renxin Wang

**Affiliations:** 1 State Key Laboratory of Dynamic Testing Technology, North University of China, Taiyuan 030051, China; 2 Department of Mechanical Engineering, Taiyuan Institute of Technology, Taiyuan 030051, China

**Keywords:** silicon nanomembrane, polydimethylsiloxane (PDMS), transfer, flexible pressure sensor, sensitivity

## Abstract

With advances in new materials and technologies, there has been increasing research focused on flexible sensors. However, in most flexible pressure sensors made using new materials, it is challenging to achieve high detection sensitivity across a wide pressure range. Although traditional silicon-based sensors have good performance, they are not formable and, because of their rigidity and brittleness, they are not suitable for fitting with soft human skin, which limits their application in wearable devices to collect various signals. Silicon nanomembranes are ultra-thin, flexible materials with excellent piezoresistive properties, and they can be applied in various fields, such as in soft robots and flexible devices. In this study, we developed a flexible pressure sensor based on the use of silicon nanomembranes (with a thickness of only 340 nm) as piezoresistive units, which were transferred onto a flexible polydimethylsiloxane (PDMS) substrate. The flexible pressure sensor operated normally in the range of 0–200 kPa, and the sensitivity of the sensor reached 0.0185 kPa^−1^ in the low-pressure range of 0–5 kPa. In the high-pressure range of 5–200 kPa, the sensitivity of the sensor was maintained at 0.0023 kPa^−1^. The proposed sensor exhibited a fast response and excellent long-term stability and could recognize human movements, such as the bending of fingers and wrist joints, while maintaining a stable output. Thus, the developed flexible pressure sensor has promising applications in body monitoring and wearable devices.

## 1. Introduction

The skin covers the surface of the body, forming the largest human organ and protecting the body from various mechanical, physical, and chemical factors. As it is in direct contact with the external environment, it can sense external stimuli, such as temperature, humidity, and pressure [1]. Inspired by the functions of human skin, researchers have developed electronic skin, which can imitate the perceptual abilities of human skin [2,3,4]. In recent years, there has been increasing research on electronic skin. Wearable devices with various functions have been developed to simulate the human body’s sensing system, such as touch and the sensing of temperature and humidity, and these devices have various applications, such as in flexible intelligent robots, medicine, and health care [5,6,7,8,9,10]. Although traditional silicon-based sensors have good performance, they are not formable because of their rigidity and brittleness and they are not suitable for applications that require fitting with soft human skin, thus limiting their applications in wearable devices [11]. Flexible sensors can conform to different surfaces of the human body without any discomfort and can also collect relevant signals to perform their sensing functions. Thus, there are new requirements for the characteristics of flexible sensors, such as low weight, low thickness, formability, and high sensitivity. Flexible tactile sensors are a crucial part of intelligent robots as they enable them to have human-like tactile perception capabilities and complete high-dexterity operations, such as grasping and releasing [12]. Pressure detection is an essential part of a sensor. To realize sensing of micro-pressure by intelligent robots, piezoresistive [13,14,15,16,17], piezoelectric [18,19,20,21], and triboelectric [22,23] sensors are used to convert external mechanical excitation into a corresponding electrical signal; these devices also exhibit excellent performance. These different sensing mechanisms have unique characteristics, which are considerably influenced by the active material and device structures. The substrate types used in contemporary flexible pressure sensors typically include materials such as polydimethylsiloxane (PDMS), polyethylene naphthalate (PEN), and polyethylene terephthalate (PET). Scholars have also fabricated structures such as pyramids and trapezoids on flexible substrates to improve device sensitivity [24]. Many new materials, such as graphene [25,26,27,28] and carbon nanotubes [29,30,31,32,33], have also been used to prepare various flexible electronic devices, such as pressure and stress sensors. The pressure sensor prepared by Chun et al. with bilayer graphene had a pressure range of 0.3 Pa–10 kPa and sensitivity of 0.24 kPa^−1^ (<250 Pa) and 0.039 kPa^−1^ (>700 Pa). Although the sensitivity was high, the detection range was relatively narrow [34]. The detection range of the three-dimensional ciliated electronic skin prepared by Lei et al. reached 200 kPa, and the sensitivity was high in the low-pressure range; however, the sensitivity decreased to 0.0008 kPa^−1^ in the range of 3–200 kPa [35]. However, these new materials are relatively expensive and cannot be easily mass produced, which limits the wide application of flexible pressure sensors. For most flexible pressure sensors, it is also challenging to achieve good sensitivity and pressure detection ranges simultaneously; thus, it is crucial to study pressure sensors that can maintain high sensitivity across a wide working range.

Microelectromechanical systems (MEMSs) are devices or systems that can be manufactured in batches and integrate microstructures, microsensors, micro-actuators, signal processing, and a control circuit. Silicon micromachining technologies mainly include silicon surface micromachining technology, silicon body micromachining technology, silicon wafer direct-bonding technology, and their mutual fusion. Design, manufacturing, packaging, reliability testing, and other common procedures help to promote the development of MEMS technology, and the market and demand for applications drives the progress in MEMS technology. With the advances in polysilicon, metal, and surface micromachining technologies, accelerometers, digital micromirrors, gas chromatographs, and resonators have developed rapidly. The subsequent development of dry-etching technology has provided an effective method for the processing of smaller devices, high-density wafer-level packaging, and depth-aspect ratio structures suitable for channel and multi-axis devices [36,37,38]. These have led to the commercialization of related developments, such as the axial-stabilized, height-aspect ratio accelerometer and the body electronic stability system (ESP), with many gyroscopic sensors being developed by Bosch, enabling consumer electronics and automobiles to sense changes in direction.

Advanced MEMS technology can feasibly be used in the fabrication and development of high-performance pressure sensors; thus, in this study, an innovative method is proposed for fabricating flexible pressure sensors based on silicon nanomembranes. First, a silicon nanomembrane (thickness = 340 nm) on the top layer of a silicon-on-insulator (SOI) wafer was skillfully transferred to PDMS as the piezoresistive material using photolithography, etching, sputtering, erosion, and transfer printing. After subsequent packaging and related equipment tests, the as-developed flexible pressure sensors operated normally in the range of 0–200 kPa and showed a sensitivity of 0.0185 kPa^−1^ in the low-pressure range of 0–5 kPa, reaching 0.0023 kPa^−1^ in the high-pressure range of 5–200 kPa. The flexible pressure sensor also exhibited excellent recoverability and fast response time and could detect motion signals of the human body, such as finger and wrist bending. Thus, the developed sensor has excellent potential for application in various fields, such as in electronic skin and human health monitoring.

## 2. Materials and Methods

### 2.1. Materials and Principle

PDMS is a colorless and transparent liquid with good chemical stability, and it can be used for a long time at temperatures between −50 and 200 °C. It is non-toxic and flexible and can be placed in contact with human skin directly; thus, it is a suitable substrate for using in flexible sensors. The PDMS used in this study was obtained from Dow Corning Corporation in the United States. The silicon nanomembrane had to be transferred twice in the experiment; therefore, it was necessary to prepare PDMS with different viscosities and different thicknesses. PDMS (Sylgard 184A) and the curing agent (Sylgard 184B) were mixed at mass ratios of 10:1 and 5:1, respectively. The different mixing ratios represent different viscosities; the higher ratio entailed a higher viscosity for the PDMS.

In semiconductors, the material is deformed after stress is applied on the surface, which changes the resistivity and resistance. This is known as the piezoresistive effect, and the change in resistance can be approximated as shown in Equation (1):

(1) $$\frac{∆R}{R}=\left(1+2\mu \right)\varepsilon +\frac{∆\rho }{\rho }\approx \frac{∆\rho }{\rho }=\pi E\varepsilon =\pi \sigma$$

where *μ* represents Poisson’s ratio, *ε* represents the axial strain of the material, *π* represents the piezoresistive coefficient of the semiconductor material in the direction of force, *E* represents the elastic modulus of the semiconductor material, and *σ* represents the axial stress acting on the material. The piezoresistive coefficient is a representative parameter that characterizes the piezoresistive effect of solid-state materials; it is the relative change in resistivity under the action of unit stress. For piezoresistive sensors, a larger piezoresistive coefficient implies a higher rate of resistance change in the varistor. At the same surface impurity concentration, the value of the piezoresistive coefficient of P-type silicon is higher than that of N-type silicon, which improves the sensitivity of the sensitive element. After comprehensive consideration, the P-type (100) SOI wafer crystal-oriented towards crystal plane <110> was selected (thickness of the top layer silicon = 340 nm, thickness of the buried oxide layer = 3 μm, thickness of the bulk silicon layer = 700 μm). Traditional silicon-based sensors are rigid and the piezoresistive material is directly processed on the SOI wafer through various procedures, making it unable to attach to a curved surface, so its applications are limited. The silicon nanomembrane for the flexible pressure sensor was obtained from a P-type SOI wafer through a series of processing methods, such as photolithography, etching, sputtering, and erosion. Then, it was successfully transferred to the flexible substrate PDMS with different viscosities and thicknesses through two transfers. After packaging, the flexible sensor had good deformation characteristics. The conductivity of silicon nanomembranes is determined by the polygonal hole concentration and the hole mobility. For bulk silicon, due to its considerable thickness, the influence of energy-band bending on the upper and lower surfaces is relatively small and can be ignored. The stress change is mainly caused by the hole mobility. However, silicon nanomembranes are extremely thin, so when the upper- and lower-surface energy bands bend and converge, the change in the hole concentration plays a dominant role, and the change in resistance at this time is greater. Therefore, the sensor prepared had good sensitivity and could provide corresponding feedback regarding the external pressure; it could also be attached to the finger joint of the human body to perceive relevant motion.

### 2.2. Fabrication of Silicon Nanomembrane

**Figure 1:**
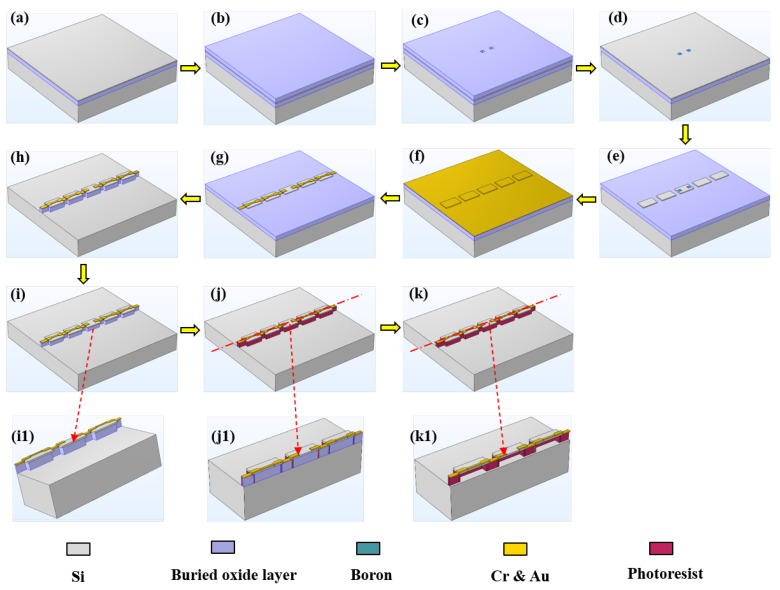
Process flowchart for silicon nanomembrane. (**a**) Cleaning wafers; (**b**) PECVD—1 μm SiO_2_ was deposited on the wafer surface; (**c**) photoetching the ohmic contact zone and etching SiO_2_; (**d**) doping boron and eroding impurities; (**e**) etching the piezoresistive region after photolithography; (**f**) sputtering metal and photolithography; (**g**) corroding metal and annealing; (**h**) etching the buried oxide layer after photolithography; (**i**) wet-etching part of the buried oxide layer; (**j**) photolithography and full exposure; (**k**) eroding the buried oxide layer; (**i1**) front view of the buried oxygen layer; (**j1**) half-section view of (**j**) along the centerline; (**k1**) half-section view of (**k**) along the centerline.

The process flowchart for the preparation of the silicon nanomembrane is shown in Figure 1, and the process was as follows:

(a) Cleaning wafers. Standard cleaning was performed on a 4 inch SOI wafer followed by drying with N_2_ to ensure that the wafer surface was clean;
(b) Plasma-enhanced chemical vapor deposition (PECVD). PECVD was performed. The SOI wafer was placed in an SI500D plasma-enhanced chemical vapor deposition apparatus produced by SENTECH company (Berlin, Germany), and 1μm thick SiO_2_ was deposited on the wafer surface;
(c) Photoetching the ohmic contact zone and etching SiO_2_. The SOI wafer was placed in a TATUNG vacuum oven to deposit hexamethyldisilazane (HMDS) adhesive at 130 °C for 1800 s and enhance the adhesion between the photoresist and the SOI wafer. The AZ6130 positive photoresist was spin-coated at 3000 rpm and then pre-baked on a hot plate at 100 °C for 75 s; then, a contact lithography machine (EVG-610TB) produced by the EVG company (St. Florian, Austria) was used for exposure. The fabricated specimen was exposed to a dose of 100 mJ/cm^2^. Then, the specimen was placed in a plasma degumming machine (ION Wave 10) at a power of 300 W for 2 min to remove the bottom film. Finally, it was placed on a drying table at 120 °C and hardened for 900 s. The SOI wafer was then placed in a reactive ion etching machine (RIE-10NR) produced by the SPTS company (Newport, England) to etch SiO_2_ at an etching rate of 20 nm/min for an estimated etching time of 50 min. After the etching was complete, the unetched silicon oxide was rinsed using the buffered oxide etch (BOE) solution. Then, an RTS-8 four-probe test system was used to measure the test area of the SOI wafer, which helped confirm whether the etched position reached the top silicon layer;
(d) Doping boron and eroding impurities. The SOI wafer was cleaned and placed in a diffusion furnace (HQ100A-3DF10) produced by Qingdao Huaqi (Qingdao, China) at 1000 °C, and concentrated boron diffusion was carried out over an ohmic contact area for 10 min. Impurities, such as silicon oxide and borosilicate glass, were formed on the surface of the top-layer silicon after the doping. Then, the wafer was placed in the BOE solution to erode at 40 °C for 480 s. After erosion, the four-probe test system was used to further confirm whether the impurities were completely corroded;
(e) Etching the piezoresistive region after photolithography. The positive photoresist was uniformly spin-coated. After exposure, the film was developed and hardened, and the piezoresistive region was retained. The reactive ion etching machine was used to etch the top-layer silicon (thickness = 340 nm) at an etching rate of 15 nm/min and etching time of approximately 23 min;
(f) Sputtering metal and photolithography. The SOI wafer was placed in an EXPLORED magnetron sputtering coater produced by the Danton Vacuum company (Philadelphia, America) and sputtered with 30 nm Cr and 300 nm Au. Then, the photoresist was evenly spin-coated to finish the photolithography progress as before and the metal area was photoetched;
(g) Corroding metal and annealing. The metals were successively corroded with the prepared gold and chromium etchants. After removing the photoresist, the SOI wafer was placed in a Hefei Kejing vacuum annealing furnace and annealed at 380 °C for 900 s so that the metal and the semiconductor formed an alloy, and the damage introduced during the diffusion process was repaired;
(h) Etching the buried oxide layer after photolithography. Considering that the thickness of the buried oxide layer was 3 μm, an AZ4620 photoresist was selected. The speed was 3000 rpm, the thickness of the photoresist was approximately 6 μm, and the exposure dose was 200 mJ/cm^2^. The SOI wafer was placed in the RIE etching machine after photolithography, and the etching time was approximately 150 min. The buried oxide layer directly under the silicon nanomembrane and the metal strip was retained, and the rest of the buried oxide layer was completely etched away;
(i) Wet-etching part of the buried oxide layer. The SOI wafer was placed in the BOE solution and eroded at room temperature. The progress in the corrosion was observed several times. After erosion, the SOI wafer was rinsed using deionized water and allowed to dry naturally. It was examined using a LEXTOLS4100 confocal microscope produced by OLYMPUS (Tokyo, Japan), and the measured corrosion depth was approximately 5 μm. A “roof structure” was formed under the piezoresistive area;
(j) Photolithography and full exposure. Full exposure was performed with an exposure dose of 20 mJ/cm^2^ after spin-coating the photoresist on the SOI wafer. After development, the film was hardened and the photoresist under the “roof structure” was retained;
(k) Eroding the buried oxide layer. The buried oxide layer was eroded using HF wet etching solution at room temperature. The progress in the corrosion was observed every few minutes. The wafer was examined under a confocal microscope after the etching was complete. The buried oxide layer was completely etched away, leaving behind the suspended silicon nanomembrane structure supported by the photoresist.

Figure 1i1 shows a front view of the buried oxygen layer, and Figure 1j1,k1 show half-section views Figure 1j,k, respectively, along the centerline.

### 2.3. Transfer of Silicon Nanomembrane

After the silicon nanomembrane structure was prepared, it was transferred to the PDMS flexible substrate. The silicon nanomembrane had to be transferred twice to the flexible substrate; thus, it was necessary to prepare PDMS with different viscosities and thicknesses. A clean 4 inch silicon wafer was placed into a PDS 2010 Parylene deposition machine produced by SCS Inc23 (Grand rapids, America) to form a 2 μm Parylene membrane to facilitate the peeling off of the PDMS from the wafer after curing. Thereafter, PDMS and the curing agent were mixed at a mass ratio of 10:1. The bubbles were extracted after even mixing and the mixture was poured uniformly onto the silicon wafer. Then, it was placed on a desktop homogenizer with different rotational speeds set to obtain PDMS with different thicknesses. Following that, the specimen was heated and cured at 75 °C for 3 h. After curing, the PDMS sample was cut into 2 × 2 cm^2^ squares, and the PDMS was slowly peeled off using tweezers and transferred to a clean silicon wafer. The PDMS thickness was tested by placing the sample in a step height measurement instrument (KLA Tencor P-7) produced by KLA Tencor company (Milpitas, CA, USA). Table 1 presents the different rotational speeds used and the different PDMS thicknesses obtained in the experiments. If the PDMS was too thick, it would not easily to attach to the surfaces of other objects, and if it was too thin, it may have resulted in cracks during the tests. We finally chose PDMS with a mass ratio of 10:1 and thickness of 350 μm as the flexible substrate. Then, we prepared PDMS with a mass ratio of 5:1 using a similar method. After curing, the PDMS was cut into strips of 1 × 5 cm^2^, and the thickness of the PDMS with the mass ratio of 5:1 was 402 μm.

**Figure 2:**
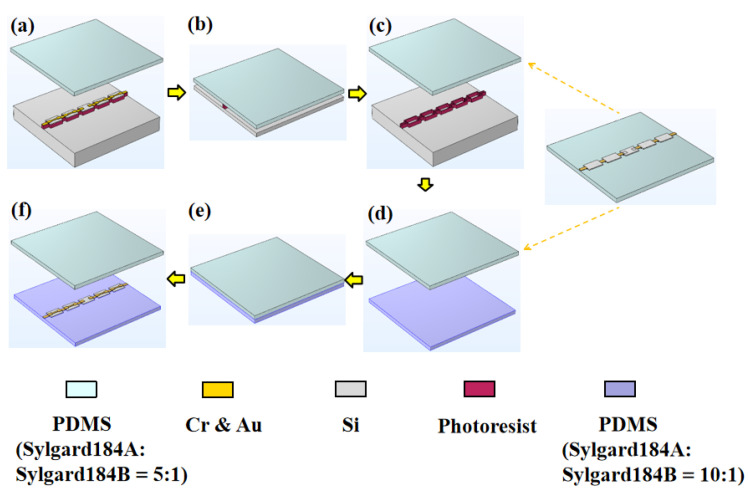
Process steps for transferring silicon nanomembrane. (**a**) The SOI wafer loaded with silicon nanomembrane was placed on a flat table; (**b**) it was covered with a long strip of PDMS with a mass ratio of 5:1 and pressed gently; (**c**) the PDMS was peeled off gently; (**d**) the PDMS loaded with the silicon nanomembrane was attached to the PDMS square with the mass ratio of 10:1; (**e**) the upward-facing PDMS was pressed gently; (**f**) the upward-facing PDMS was peeled off gently, and then the silicon nanomembrane was transferred to the downward-facing PDMS.

The steps of the process of transferring the silicon nanomembrane are shown in Figure 2. The SOI wafer loaded with the silicon nanomembrane was placed on a flat table (Figure 2a). It was covered with a long strip of PDMS with a mass ratio of 5:1 and pressed gently with the thumb (Figure 2b). The PDMS was gently peeled off and the SOI wafer was left with a ring of supporting photoresist (Figure 2c). The PDMS sample was observed using a confocal microscope to determine whether the transfer was successful. After the first successful transfer printing, the PDMS loaded with the silicon nanomembrane (note that the side of the silicon nanomembrane was placed upward) and the PDMS square with a mass ratio of 10:1 was positioned on a clean silicon wafer, and then it was placed in an oxygen plasma degumming machine for pretreatment. The power was set to 300 W, the time was 10 s, and the pressure was 150 mTorr. The pretreatment was conducted to realize the bonding of the PDMS and the silicon nanomembrane. After pretreatment, the silicon wafer was placed on a horizontal table, and the PDMS with the mass ratio of 5:1 loaded with the silicon nanomembrane was attached to the PDMS square with the mass ratio of 10:1 (Figure 2d). The upward-facing PDMS with the mass ratio of 5:1 was pressed gently (Figure 2e). It was then peeled off gently, and the silicon nanomembrane was transferred to the PDMS with the ratio of 10:1 (Figure 2f). After the secondary transfer, the specimen was examined again under a confocal microscope to confirm that the sample was intact.

### 2.4. Integration of the Sensor

**Figure 3:**
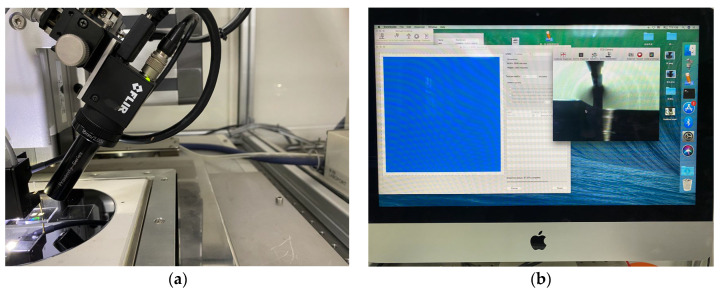
High-precision micro-nano-material deposition system for printing silver electrodes. (**a**) Enlarged view of the silver electrode printed on the PDMS flexible substrate; (**b**) host real-time display when the electrodes were printed on both sides of the silicon nanomembrane.

After the silicon nanomembrane was successfully transferred to the PDMS flexible substrate, electrodes were drawn. We used a high-precision micro-nano-material deposition system (Sonoplot Microplotter II) produced by SonoPlot Inc (Middleton, WI, USA). Ag ink was directly printed on flexible substrates using inkjet deposition. To enhance the adhesion between the Ag ink and the PDMS flexible substrate, the PDMS had to be pretreated with oxygen plasma before printing at a power of 300 W for 120 s. Then, the silicon wafer containing the sample was placed on the workbench of the system, and the heating switch of the workbench was turned on in advance, which ensured that the temperature could reach 80 °C before printing and was helpful for curing the Ag ink during the printing process. A needle with a diameter of 10 μm was installed at the designated position, and a small amount of Ag ink was drawn with a dropper and put into a small container for later use. Figure 3a shows an enlarged view of the silver electrodes printed on the PDMS flexible substrate. A needle was moved using the corresponding software operation to determine the position of the silicon nanomembrane on the screen and the coordinates were recorded; then, it was returned to the small container to absorb the Ag ink. The printing was started when the needle reached the edge of the silicon nanomembrane side, as shown in Figure 3b. Electrodes with a size of 5 × 5 mm^2^ were drawn in advance using the drawing function and printed several times to ensure optimal conductivity. After printing, the silicon wafer was immediately placed on a drying table at 120 °C for 1 h to cure the Ag ink.

After curing, two wires were placed on both sides of the printed Ag electrodes, and then a small amount of conductive silver paste was applied. The specimen was then heated and cured for 20 min. Conductive tape was attached to the top of the silver electrode to fix the wire, and then the prepared PDMS solution was poured on the top of the sample and the sample was heated on a drying table at 75 °C for 3 h. After the PDMS was cured, the excessive PDMS around the sample was cut away to complete the sensor packaging.

## 3. Results and Discussion

### 3.1. Characterizations of Silicon Nanomembrane

**Figure 4:**
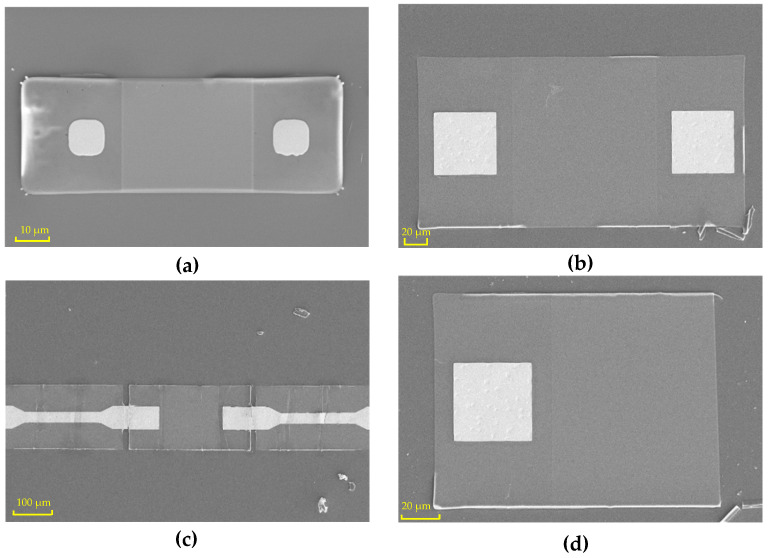
Characterization of silicon nanomembranes on PDMS flexible substrate after the second transfer. (**a**) Morphological features of silicon nanomembrane with a size of 100 × 40 μm^2^; (**b**) morphological feature of silicon nanomembrane with a size of 300 × 160 μm^2^; (**c**) morphology feature map of the silicon nanomembrane with continuous metal strips on both sides and middle silicon nanomembrane size of 300 × 160 μm^2^; (**d**) morphology feature map of the broken silicon nanomembrane after the transfer.

When the silicon nanomembrane was prepared, a field emission scanning electron microscope (SEM; SUPRA-55) from ZEISS company (Oberkochen, Germany) was used to characterize the samples. After heavy doping, etching, sputtering, erosion, and other processes, the silicon nanomembrane was ready. Following two transfers, the silicon nanomembrane was successfully transferred to the PDMS flexible substrate. Figure 4 shows the topography of the silicon nanomembranes on the PDMS after the second transfer. Silicon nanomembranes of different sizes were designed on the mask. One size was 100 × 40 μm^2^, and the side length of the metal area was 12 μm. The actual size of the silicon nanomembrane after the transfer was 95.83 × 34.28 μm^2^. The side length of the metal region was 10.54 μm, as shown in Figure 4a. Another one of the silicon nanomembranes designed had a size of 300 × 160 μm^2^, and the side length of the metal area was 60 μm. After the transfer, the measured size was 293.2 × 153 μm^2^, and the side length of the metal area was 55.57 μm, as shown in Figure 4b. The actual measured size was slightly different from the designed size, which may have been due to a slight deviation in the preparation process, such as during etching. Due to the small size of the silicon nanomembrane, it could not be easily aligned when printing the electrodes. Therefore, continuous silicon nanomembranes were designed with metal strips on both sides in the middle position. The metal strips covered the top of the silicon nanomembrane. When the PDMS transferred the silicon nanomembrane, it also transferred the metal strips attached to it. After the transfer, the metal strips on both sides of the silicon nanomembrane were intact, as shown in Figure 4c. After both of the transfers, some silicon nanomembranes were damaged, as shown in Figure 4d. Only the left half of the silicon nanomembrane was transferred successfully after the two transfers, while the middle of the silicon nanomembrane was fractured in the transfer process, so the right half was lacking. The silicon nanomembranes were not completely removed from the buried oxide layer due to the incomplete corrosion.

### 3.2. Sensing Performance of the Flexible Pressure Sensor

**Figure 5:**
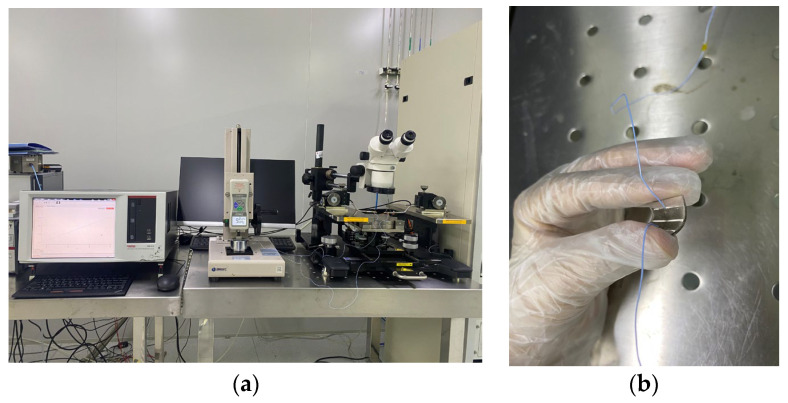
Images showing the physical testing of the flexible pressure sensor. (**a**) Flexible pressure sensor under 9 N pressure; (**b**) flexible pressure sensor.

To test the performance of the flexible pressure sensor, we placed the packaged sensor on the lower bracket of a pressure testing machine and connected the wires on both sides of the sensor to the two probes of an EPS150TRIAX normal temperature probe station produced by the Suzhou Yioulu system integration company (Suzhou, China), which was connected to both ends of a 4200-SCS semiconductor analyzer (KEITHLEY). The pressure testing machine was used to apply different levels of pressure to the flexible sensor, and the resistance value of the flexible sensor under different levels of pressure was tested with the semiconductor analyzer. The sensitivity was calculated using Equation (2):

(2) $$S=\frac{\left(R-R_{0}\right)/R_{0}}{∆P}$$

where *R*_0_ represents the initial resistance value of the flexible sensor when no pressure was applied, *R* represents the resistance value of the flexible sensor after applying pressure, and Δ*P* represents the change in pressure. To test the performance of the flexible sensor under different levels of pressure, we used two pressure testing machines (ZA-21A-2 and ZQ-32) produced by the Zhiqu precision instrument company (Dongguan, China). Their measurement limits were 10 N and 500 N, and the accuracies were 0.01 N and 1 N, respectively. Images of the physical testing of the flexible pressure sensor based on silicon nanomembranes are shown in Figure 5.

**Figure 6:**
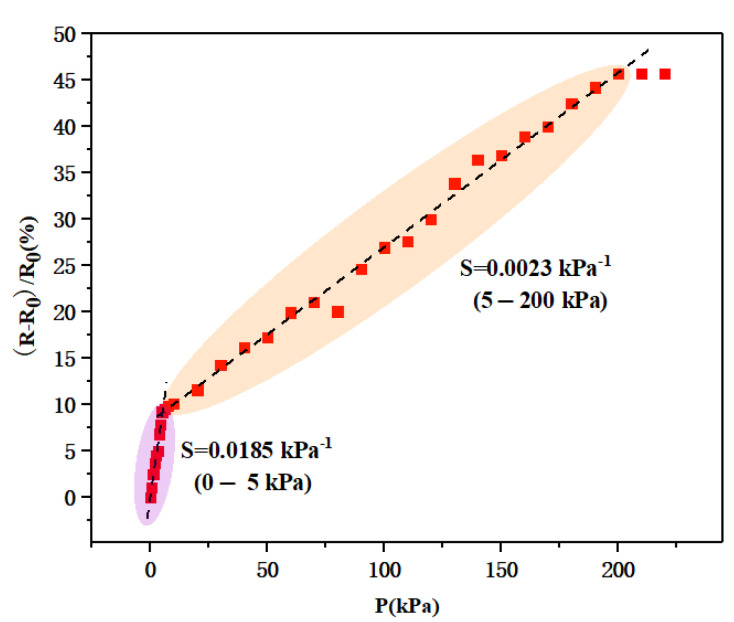
The sensitivity curve for the flexible pressure sensor based on silicon nanomembranes.

The sensitivity curve for the flexible pressure sensor based on silicon nanomembranes is shown in Figure 6. The sensor worked normally in the pressure range of 0–200 kPa, and it exhibited a sensitivity of 0.0185 kPa^−1^ in the low-pressure range (0–5 kPa) and 0.0023 kPa^−1^ in the high-pressure range (5–200 kPa). The silicon nanomembrane was prepared from the top layer of the SOI wafer through a series of processes; therefore, the thickness was only 340 nm, and it had a certain flexible deformation ability after it was transferred to the PDMS flexible substrate. The resistance of the film changed significantly and the sensitivity was relatively high when we started applying pressure. As the applied pressure gradually increased, the resistance of the silicon nanomembrane changed slowly, and the sensitivity in the range of 5–200 kPa was not as high as that in the range of 0–5 kPa. We also determined that, when the applied pressure exceeded 200 kPa, the resistance of the flexible pressure sensor changed only slightly, indicating that the deformation of the silicon nanomembrane was close to saturation.

**Figure 7:**
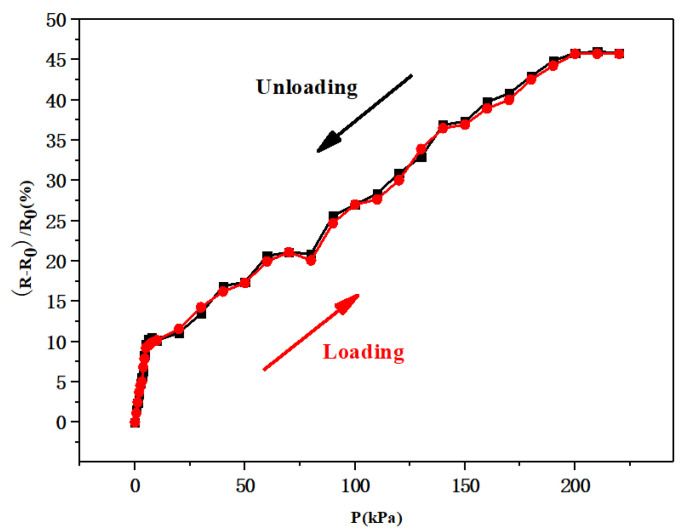
Relative resistance variation for the flexible pressure sensor during pressure loading and unloading.

Recoverability is an essential factor that must be considered when evaluating the performance of flexible pressure sensors for practical applications. Figure 7 shows a comparison of the relative resistance variations for the flexible pressure sensor under different loading (red curve, 0–200 kPa) and unloading pressures (black curve, 200–0 kPa). These two curves were approximately the same, indicating that the prepared flexible pressure sensor could quickly recover to the initial state once the applied pressure was released, thus verifying the good recoverability of the sensor.

**Figure 8:**
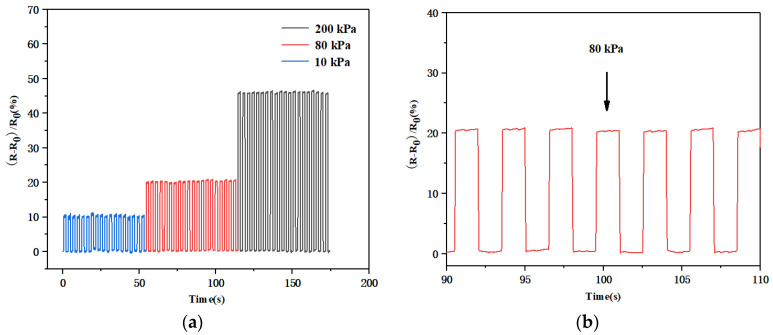
(**a**) Resistive response of the sensor under different periodic pressures (10, 80, and 200 kPa); (**b**) the resistive response of the sensor at 80 kPa.

The periodic response, long-term stability, detection limit, and response time are all crucial indicators when measuring the performance of sensors. First, we studied the stability of the pressure response of the sensor and recorded the relative variation in the resistance of the flexible pressure sensor under different periodic pressures (10, 80, 200 kPa), as shown in Figure 8. As the applied pressure increased, the variation in the resistance of the flexible pressure sensor gradually increased, presenting a step-like change. Figure 8b represents the response of the resistance at 80 kPa. Under a certain fixed pressure, a relatively stable square wave was obtained, indicating that the sensor was sensitive to different pressures and could provide corresponding pressure feedback.

**Figure 9:**
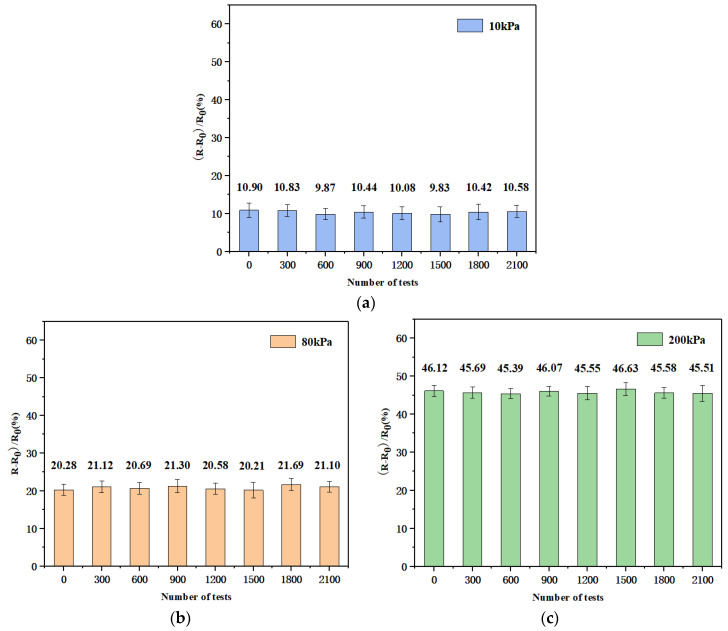
Variation in the relative resistance of the flexible pressure sensor at pressures of 10, 80, and 200 kPa after 0, 300, 600, 900, 1200, 1500, 1800, and 2100 cycles, the error bars represent the standard deviations for the experimental data; (**a**) variation in the relative resistance of the sensor at 10 kPa; (**b**) variation in the relative resistance of the sensor at 80 kPa; (**c**) variation in the relative resistance of the sensor at 200 kPa.

Furthermore, applying and releasing pressure multiple times can degrade the performance of flexible sensors, especially in environments with harsh working conditions, which inevitably affects the accuracy of the measured signal. Therefore, we conducted further research on the long-term stability of the sensor. The change in the resistance was examined when different pressures were continuously and periodically applied for more than 2000 cycles. Figure 9 shows the variation in the relative resistance of the flexible pressure sensor at pressures of 10, 80, and 200 kPa after 0, 300, 600, 900, 1200, 1500, 1800, and 2100 cycles. It shows that the resistance of the sensor did not change significantly, indicating that the sensor had good durability and cycle stability.

**Figure 10:**
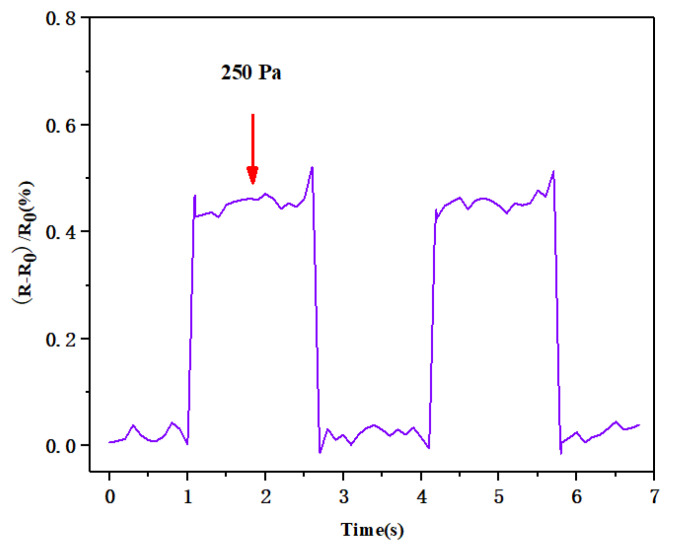
Real-time response test of the flexible pressure sensor for repeated loading/unloading of low pressure (250 Pa).

After testing, we found that the minimum detection level of the flexible pressure sensor was 0.1 N, and the corresponding pressure was 250 Pa, as shown in Figure 10. The sensor had a sensitivity of 0.0185 kPa^−1^ in the pressure range of 0–5 kPa; when the applied pressure was <250 Pa, the resistance of the sensor could not be detected, so the minimum detection limit of this flexible pressure sensor was 250 Pa.

**Figure 11:**
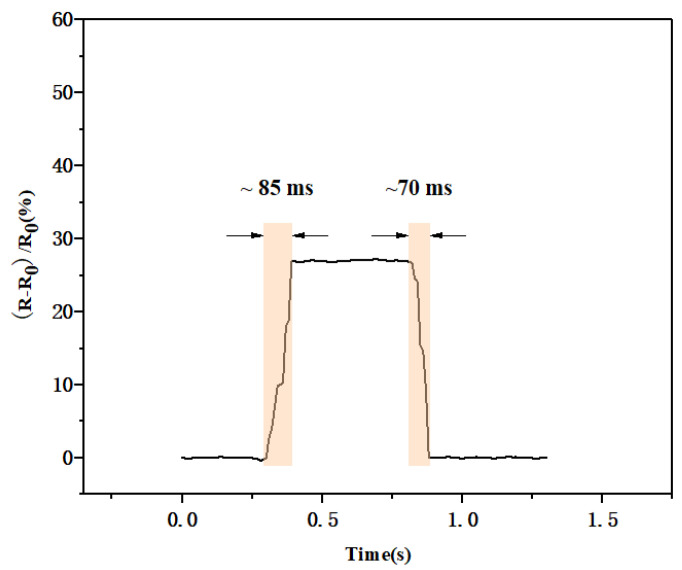
Response time of the flexible pressure sensor when exposed to external pressure.

The response time of the sensor is also an important index for evaluating sensor performance. Figure 11 shows the response time of the sensor under external pressure of 100 kPa. The sensor only needed 85 ms after the external pressure was applied to generate a stable waveform, and it required 70 ms to restore the initial state, which also indicated its excellent response time.

The above tests showed that the prepared flexible pressure sensor exhibited good performance, and a comparison of its parameters with existing pressure sensors is shown in Table 2. The detection range of the prepared flexible pressure sensor reached 200 kPa, and it maintained good sensitivity across a wide pressure range. It also had good recoverability and fast response. The excellent performance highlighted its potential to reliably detect pressure with perceptual feedback in various applications.

### 3.3. Application of the Flexible Pressure Sensor

**Figure 12:**
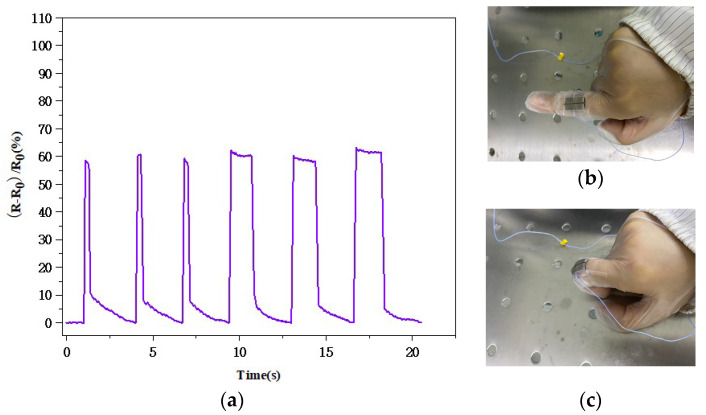
(**a**) Real-time response graph for the flexible pressure sensor attached to the finger joint; (**b**) the finger joint in a straight state; (**c**) the finger joint in a bending state.

The PDMS substrate was selected for the flexible pressure sensor because it can attach well to the surface of the human body to monitor various signals. To test the detection ability of the sensor with different mechanical stimuli, we attached it to the index finger of a volunteer. Figure 12 shows the real-time response of the flexible pressure sensor when the finger was bent. The bending of the finger joints deformed the sensor substrate and changed the resistance of the silicon nanomembrane; therefore, we observed a regular waveform when the finger was bent.

**Figure 13:**
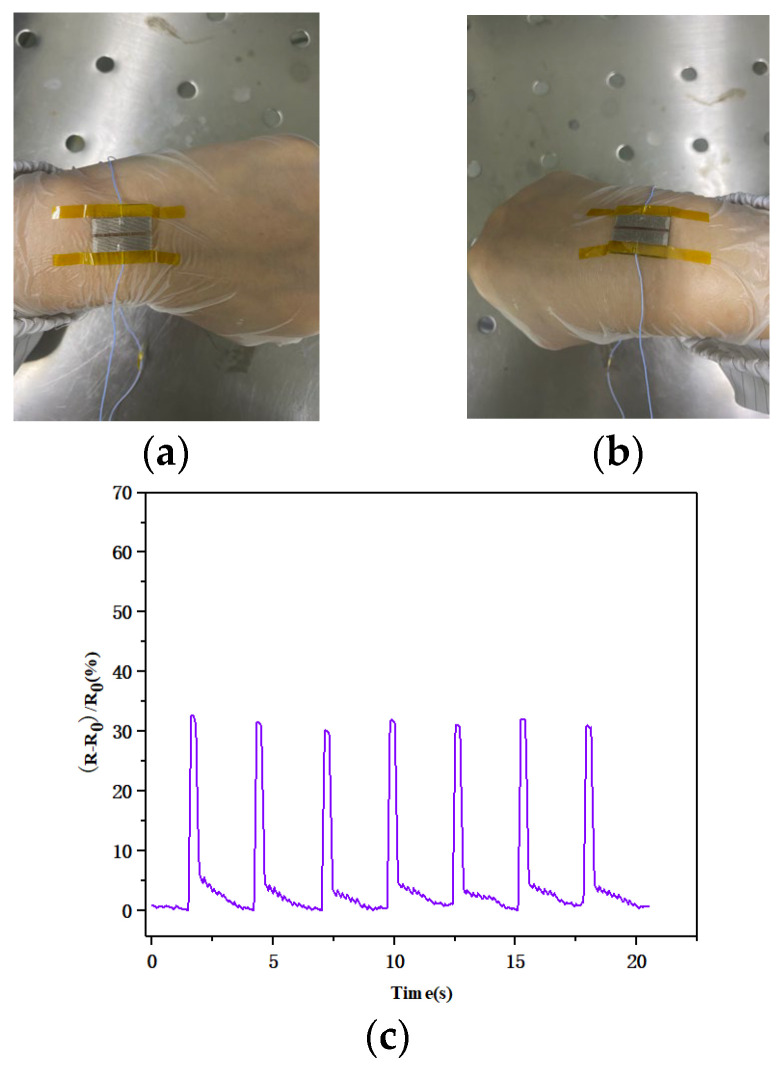
(**a**) The wrist joint in a straight state; (**b**) the wrist joint in a bending state; (**c**) real-time response graph for the flexible pressure sensor attached to the wrist.

We also attached the flexible pressure sensor to a human wrist to further test the perception function of the sensor. As shown in Figure 13, the resistance of the flexible pressure sensor did not change when the wrist was not bent. The morphology of the silicon nanomembrane changed slightly due to bending, which changed its resistance. The relative rate of change in the resistance of the flexible pressure sensor was basically between 30.18 and 32.17% when the wrist was constantly bent, and there was a slight difference each time because the degree of wrist bending was not completely identical, which indicated that the flexible pressure sensor could maintain a relatively stable output. Tiny mechanical strengths and stimuli were accurately recorded by the flexible pressure sensor, and the repetitive and stable waveform also clearly indicated that the sensor effectively identified the bending of wrist joints. These results show that the sensor has great application potential in the monitoring and recording of human motion, providing novel insights for designing wearable devices.

## 4. Conclusions

In this study, we developed a method for preparing flexible pressure sensors based on silicon nanomembranes. The silicon nanomembrane structure was prepared through a series of processes, such as photolithography, etching, heavy doping, sputtering, and erosion. After preparing PDMS with different viscosities and thicknesses, the silicon nanomembrane was successfully transferred to the PDMS flexible substrate, and the materials were encapsulated and fabricated into a flexible pressure sensor. After testing, the sensor operated in the pressure range of 0–200 kPa and showed sensitivities of 0.0185 kPa^−1^ (0–5 kPa) and 0.0023 kPa^−1^ (5–200 kPa). The response time was only 85 ms, and the repeatability and durability were excellent. The PDMS flexible substrate enabled it to better fit the human skin, and it could sense human motion signals accurately when the finger and wrist joints were bent. Thus, the developed flexible pressure sensor holds promise for application in electronic skin and wearable devices.

## Figures and Tables

**Table 1:** Different rotational speeds corresponding to different PDMS thicknesses.

Number	Low Speed (rpm)	Constant Time (s)	High Speed (rpm)	Constant Time (s)	Measured Thickness (μm)
1	500	10	1500	15	83
2	500	10	1200	15	103
3	500	10	1000	10	159
4	500	10	800	10	169
5	500	10	750	10	193
6	200	10	0	0	350
7	100	10	0	0	402
8	10	3	0	0	502

**Table 2:** Comparison of the fabricated flexible pressure sensor and existing pressure sensors.

Source	Substrate	Sensitive Material	Transduction Principle	Sensitivity (GF)	Range
Chun, S. et al. [17]	PET	Double-layered graphene	Piezoresistivity	0.24 kPa^−1^ (<250 Pa)	0.3 Pa–10 kPa
				0.039 kPa^−1^ (>700 Pa)	
Lei, M. et al. [35]	PDMS/CIP	Carbon-based conductive materials	Piezoresistivity	0.0198 kPa^−1^ (<3 kPa)	0 Pa–200 kPa
				0.0008 kPa^−1^ (3 kPa–200 kPa)	
Zhang, J. et al. [39]	Micro-pyramid PDMS	Reduced graphene oxide (RGO)	Piezoresistivity	−1.71 kPa^−1^ (<2 kPa)	0 Pa–5 kPa
				−0.02 kPa^−1^ (2–5 kPa)	
Smith., A.D. et al. [40]	Cavities etched into a SiO_2_ film on a silicon substrate	Graphene membranes	Piezoresistivity	2.25 × 10^−3^ kPa^−1^	0 Pa–100 kPa
Zhang, J.H. et al. [12]	PDMS/epoxy pillars	PVDF	Piezoelectricity	346.9 pCN^−1^	0.009–4.3 N
Chun, S. et al. [34]	PEN	CNT sheets	Capacitance	0.06–0.13% (<20 kPa)	1 Pa–40 kPa
				0.02–0.04% (20–40 kPa)	
This work	PDMS	Silicon nanofilm	Piezoresistivity	0.0185 kPa^−1^ (<5 kPa)	0–200 kPa
				0.0023 kPa^−1^ (5–200 kPa)	

## Data Availability

Not applicable.
